# Absolute and Relative Depth-Induced Network for RGB-D Salient Object Detection

**DOI:** 10.3390/s23073611

**Published:** 2023-03-30

**Authors:** Yuqiu Kong, He Wang, Lingwei Kong, Yang Liu, Cuili Yao, Baocai Yin

**Affiliations:** 1 School of Innovation and Entrepreneurship, Dalian University of Technology, Dalian 116024, China; yqkong@dlut.edu.cn (Y.K.);; 2 School of Computer Science and Technology, Dalian University of Technology, Dalian 116024, China; 3 School of Information and Communication Engineering, Dalian University of Technology, Dalian 116024, China

**Keywords:** RGB-D salient object detection, multi-modal analysis and understanding, multi-modal fusion strategy

## Abstract

Detecting salient objects in complicated scenarios is a challenging problem. Except for semantic features from the RGB image, spatial information from the depth image also provides sufficient cues about the object. Therefore, it is crucial to rationally integrate RGB and depth features for the RGB-D salient object detection task. Most existing RGB-D saliency detectors modulate RGB semantic features with absolution depth values. However, they ignore the appearance contrast and structure knowledge indicated by relative depth values between pixels. In this work, we propose a depth-induced network (DIN) for RGB-D salient object detection, to take full advantage of both absolute and relative depth information, and further, enforce the in-depth fusion of the RGB-D cross-modalities. Specifically, an absolute depth-induced module (ADIM) is proposed, to hierarchically integrate absolute depth values and RGB features, to allow the interaction between the appearance and structural information in the encoding stage. A relative depth-induced module (RDIM) is designed, to capture detailed saliency cues, by exploring contrastive and structural information from relative depth values in the decoding stage. By combining the ADIM and RDIM, we can accurately locate salient objects with clear boundaries, even from complex scenes. The proposed DIN is a lightweight network, and the model size is much smaller than that of state-of-the-art algorithms. Extensive experiments on six challenging benchmarks, show that our method outperforms most existing RGB-D salient object detection models.

## 1. Introduction

**Figure 1:**
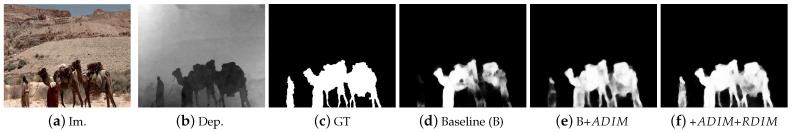
Effectiveness of absolute depth information and relative depth information. (**a**) Input image; (**b**) depth image; (**c**) ground truth; (**d**) saliency map generated by baseline network; (**e**) saliency map using absolution depth information; (**f**) saliency map using both relative depth information and absolute depth values.

Salient object detection aims to locate and segment the most visually distinctive objects in an image. It often serves as a pre-processing method, that focuses the attention on semantically meaningful regions and provides informative visual cues to the downstream tasks, such as scene classification [1,2], visual tracking [3,4], foreign object detection [5], etc. In recent years, the rapid development of convolutional neural networks (CNNs) has facilitated the significant improvement of various computer vision tasks, e.g., object detection [6], brain tumor segmentation [7], and point cloud segmentation [8]. Salient object detection also benefits from the powerful representation ability of CNNs [9]. However, many problems still exist to be solved to accurately detect salient objects in complex image scenes, such as similar appearances between foreground and background areas, low-intensity environments, multiple objects of different sizes, etc. Due to the limited representation ability of existing saliency models, it is challenging to discriminate salient objects from cluttered background regions with only RGB images. Recently, benefiting from the development of depth sensors, it has been convenient to obtain dense depth images. Pixel-wise depth values, provide spatial information and geometric cues of the scene, which are complementary to the appearance features of the RGB data. Compared with only using appearance features from RGB images, saliency models based on RGB-D cross-modalities, can capture more relevant information about objects and avoid redundant noise. For example, in the very complex scene in Figure 1, where salient objects are not distinctive in the local area, we can observe obvious contrast between the foreground and background regions in the depth space (Figure 1b).

This paper proposes a cross-modal fusion strategy for RGB-D salient object detection. Specifically, we consider two kinds of depth information, pixel-wise absolute depth values from depth images and relative depth values (also known as spatial distance in 3D space) between pair-wise pixels. The interaction between absolute depth information and RGB images is a hot topic for scene understanding, such as person re-identification [10], 3D object detection [11,12,13], etc. Most existing methods learn to extract depth and RGB features by separate networks, and directly fuse them in each scale using different fusion modules [14,15,16], ignoring the message transmission across different scales. Moreover, because of the large gap between the distribution of RGB and depth data, it will inevitably introduce noisy responses in prediction results. For example, the saliency map in Figure 1d, which is generated by the baseline network (simply integrating RGB and depth features with concatenation and convolution blocks), cannot effectively capture the entire salient object in the scene. As comparison, the network with the proposed ADIM (Figure 1e) can respond to most salient regions, by reasonably combining RGB-D cross-modal features. Besides the information provided by absolute depth values, we argue that relative depth values between short-range pixels also contribute to recognizing distinctive saliency cues in the local area. However, these have rarely been studied in recent work. For example, the left person in Figure 1a is less discriminative in the RGB feature space, but presents obvious disparity around the object boundary in the depth space. Therefore, by introducing relative depth information, the proposed method manages to recognize the person as a salient object (Figure 1f).

To this end, we propose a depth-induced network (DIN) for RGB-D salient object detection, which consists of two main components, the absolute depth-induced module (ADIM) and the relative depth-induced module (RDIM). Unlike directly fusing features from the depth and RGB images, the ADIM enforces in-depth interaction of the two modalities in a coarse-to-fine manner. Specifically, a set of gate recurrent units (GRU) [17] are employed, to hierarchically integrate depth and RGB features across multiple scales. The gate structure adaptively selects informative features from the RGB and depth images, and thus controls the fusion of multi-modalities and avoids cluttered noise being introduced, caused by the asynchronous properties of the two feature spaces. The ADIM is implemented in a recursive manner. The fusion results in the shallower layer, are subsequently input into the next integration step in the deeper layer, ensuring effective information transfer across different scales. By this means, the degree of integration goes deep through the network, and we can explore saliency cues from the combined features at different scales. The RDIM aims to capture the spatial structure information of the image and detailed saliency cues, by utilizing relative depth information. Since adjacent pixels in 2D space may not be strongly associated with 3D point cloud space, we project image pixels into 3D space based on their spatial positions and depth values. Then, a graph model is constructed on the feature map level, to enforce information propagation in the local area, according to the relative depth relationship. We implement it by a spatial graph convolutional network (GCN), based on the relative depth and semantic affinities between pair-wise pixels. The feature representation ability is successively enhanced by exploring spatial structure and geometry information across multi-scales. By this means, detailed saliency cues are exploited by the RDIM, which facilitates the accurate prediction of the final results. Unlike the commonly used two-stream networks, which encode the RGB and depth images with the same architecture [18,19,20,21], the proposed DIN is a single-stream network. It thus dramatically reduces the computation costs without sacrificing the model performance.

In summary, the main contributions of this work are three-fold:

- We propose an ADIM which adopts the GRU-based method and adaptively integrates absolution depth values and RGB features, to combine the geometric and semantic information from multi-modalities.
- We propose an RDIM which employs spatial GCN to explore semantic affinities and contrastive saliency cues, by leveraging the relative depth relationship.
- The proposed DIN for RGB-D salient object detection is a lightweight network and outperforms most state-of-the-art algorithms on six challenging datasets.

## 2. Related Works

This section reviews some representative works on salient object detection and RGB-D salient object detection, respectively. We also give a brief discussion on the graph convolutional network.

### 2.1. Salient Object Detection

Early salient object detection approaches [22,23,24,25] mainly used hand-crafted features, such as brightness, color, and texture, to locate and segment salient objects in an image. In recent years, thanks to the development of CNNs, various deep learning-based saliency models [26,27,28] have been proposed, and they outperform the traditional methods by a large margin. Zhang et al. [26], explored multi-level features in each scale and recursively generated saliency maps. Feng et al. [27], proposed an attentive feedback module to explore the structure of salient objects better. Kong et al. [28], designed propagation modules to combine multi-scale features of the network. The authors of [29], proposed an integrity cognition network, to enhance the integrity of the predicted salient objects. In [30], a dual graph model was established, to guide the focal stack fusion for light field salient object detection. The authors of [31], developed a salient object detector without any human annotations by a novel supervision synthesis scheme.

Although the above works study various multi-scale fusion models and learning strategies, they still face challenges where the scenarios are complicated. To address this issue, we resort to depth images to explore the spatial structure and geometric information of the scene, and thus improve the effectiveness and robustness of the network.

### 2.2. RGB-D Salient Object Detection

Traditional hand-made RGB-D salient object detection methods [32,33,34] have inferior representation ability of semantic and geometric features. Recently, CNN-based methods have been developed, that are powerful in modeling the RGB-D multi-modalities and thus improve the detection performance to a large extent. Effectively integrating depth and RGB features is one of the critical issues in this task. In [35], Piao et al. hierarchically integrates the depth and RGB image, and refines the final saliency map by a recurrent attention model. The work [19], designed an asymmetric two-stream network for learning the multi-scale and multi-modal features by a ladder-shape module and attention strategies. Sun et al. [20], explored the depth-wise geometric prior, to refine the RGB feature, and employed automatic architecture search to improve the performance of the saliency model. To reduce the model size and improve the performance, Zhao et al. [36] proposed a one-stream network for RGB-D salient object detection and designed effective attention models to combine multi-modalities. The work [37], proposed a novel mutual attention model, to fuse cross-modal information. Besides, effective learning strategies are also crucial for a high-quality detection model. Ji et al. [38], proposed a novel collaborative learning framework, to enhance the interaction of edge, depth, and saliency cues. The authors of [18], trained a depth distiller, which modulated the RGB representation by the features in the depth stream. In [39], two kinds of self-supervised pre-training processes were conducted, to learn semantic information and reduce the inconsistency between multi-modalities.

The mutual learning method was employed in [40,41], to align RGB and depth features. Liu et al. [42], proposed a unified transformer architecture for both RGB and RGB-D salient object detection, to propagate global information among image patches. The work [43], proposed a transformer-based network, to learn implicit class knowledge for RGB-D co-saliency detection. Although saliency models can benefit from depth information and distinguish objects from cluttered scenes, sometimes depth images are inaccurate or hard to obtain, thus introducing inevitable noise in the prediction results. Considering this situation, Hussain et al. [44] proposed to leverage only RGB images during both training and test stages, first predict depth values, and generate final saliency maps based on the intermediate depth information. This method employed a combination of Transformer and CNN, leading to satisfactory predicting results. Most of these methods exploit discriminative cues from the absolute depth values but ignore the structure information indicated by the relative depth values. In our work, we make full use of both absolute and relative depth information in images, with a single-stream architecture, to facilitate the accurate saliency detection of RGB images.

### 2.3. Graph Convolutional Network

A GCN aims to learn geometry information on non-Euclidean structural data. Because of the flexible application of the relationship between nodes, GCNs have received more and more research interest in recent years. Generally speaking, GCNs can be categorized into spectral approaches and spatial approaches. Kipf et al. [45], proposed a spectral GCN which performed the convolution operation in the spectral domain on the constructed graphs, with the help of Fourier transformation. Velickovic et al. [46], designed a spatial GCN that fused features between neighbor nodes with an attention mechanism. A multi-layer perceptron was trained to learn the affinity relationship between adjacent nodes. Battaglia et al. [47], utilized multiple blocks to update and transfer information on the graph alternately. Due to the effectiveness of GCNs, many researchers employ them in computer vision tasks. Yao et al. [48], used a GCN to integrate both semantic and spatial relationships of objects, to generate a more accurate image caption. Qi et al. [49], represented the image as a graph model with the depth prior. By constraining the GCN with invisible depth information, a more accurate image segmentation result is obtained. In ref. [50], a GCN is employed to model the semantic relationship on both language and visual modalities and then infers the referred regions in the image. Inspired by these works, we design a spatial GCN for the RGB-D saliency detection task. This network fully uses relative depth information, to explore detailed saliency cues in the local area and obtains satisfactory detection results with much clearer object boundaries.

## 3. Algorithm

We propose a DIN that leverages depth images to induce spatial relationships for RGB saliency detection. We first introduce the overall architecture in Section 3.1. We discuss the ADIM, which fuses the image and absolute depth features, in Section 3.2, and we elaborate on the RDIM, which refines the fused features based on the guidance of relative depth information, in Section 3.3.

### 3.1. Overall Architecture

**Figure 2:**
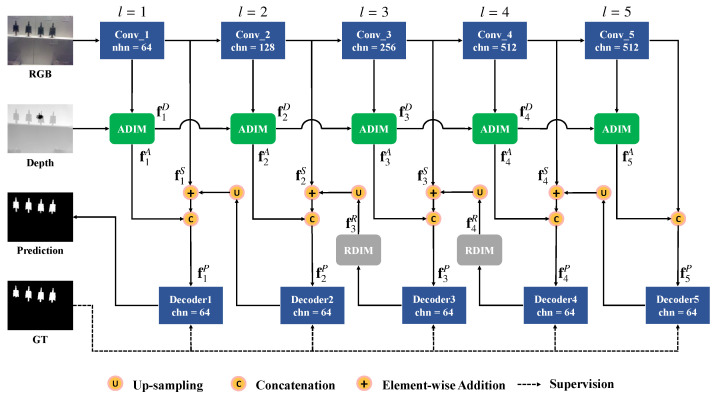
Architecture of the proposed DIN model. There are three main components in the network: the backbone network; the ADIM, which fuses RGB and depth images in the encoding stage; and the RDIM, which complements relative depth cues in the decoding stage.

The overall architecture of the proposed DIN is shown in Figure 2. We employ a ResNet [51]-based network as the backbone, to encode the input RGB image. The backbone network consists of five convolution blocks, including Conv_1, Conv_2,⋯, Conv_5. Fed with an RGB image *I*, with size W×H, it generates multi-scale feature maps $f_{l}^{I}$ with size $\frac{W}{2^{l-1}}\times \frac{H}{2^{l-1}},l=1,2,\cdots ,5$, respectively. We removed the fully connected layers of the original ResNet [51] to fit this task. The depth image *D*, is first encoded with a set of convolutional layers and then successively warped and integrated with the hierarchical image feature maps by the proposed ADIM, generating the fused feature maps $f_{l}^{A},l=1,2,\cdots ,5$.

Since features from different levels represent meaningful information, we recursively integrate multiple feature maps in the decoding stage. Specifically, the image feature maps $f_{l}^{I}$ are first fed into a convolutional layer with kernel size 1×1, to reduce the channel number to 64, and the generated side output feature maps are denoted as $f_{l}^{S},l=1,2,\cdots ,5$. Then the multi-level feature maps of RGB and depth images are integrated in a top-down manner,

(1) $$f_{l}^{P}=\left\{\begin{array}{cc} ReLU(Conv\left(C\left(f_{l}^{A},f_{l}^{S}+U\left(f_{l+1}^{P}\right)\right)\right), & l=1,2,\cdots ,4\\ ReLU(Conv\left(C\left(f_{l}^{A},f_{l}^{S}\right)\right)), & l=5 \end{array}\right.$$

in which ReLU(·) is the ReLU activation function, Conv(·) is the convolution layer, C(·,·) is the concatenation operation that concatenates feature maps on the channel dimension, and U(·) is the up-sampling operation with bilinear interpolation. + represents the element-wise addition operation. The feature map $f_{l}^{P}$, denotes the integrated RGB-D features in the *l*-th scale.

To further exploit the spatial structure information and detailed saliency cues, we refine the integrated feature maps $f_{l}^{P}$, with the proposed RDIMs, by considering the relative depth values. The output feature maps are denoted as $f_{l}^{R}$. To balance the computation costs and performance, we apply the RDIM on the 3rd and 4th-levels. Then, the integrated feature maps are fed into a set of convolutional layers, with kernel size 3×3, to generate saliency maps $S_{l},l=1,2,\cdots ,5$, at multiple scales. The proposed depth-induced network (DIN) is trained in an end-to-end manner. All the saliency maps are directly supervised by the ground truth, and the losses are summarized and optimized jointly. Considering that the feature maps $f_{1}^{P}$, incorporate high-level semantic information and low-level detailed knowledge, we choose $S_{1}$ as the final prediction result.

### 3.2. Absolute Depth-Induced Module

The goal of the absolute depth-induced module (ADIM), is to integrate appearance features from the RGB image and depth information from the depth image. Since there is a large gap between the distribution of RGB and depth data, flat feature fusing methods will introduce cluttered noise in the final prediction. Therefore, we propose to recursively fuse features of the two modalities, by employing a series of ADIMs to enforce the in-depth interaction between RGB and depth features. As shown in Figure 2, the depth information is embedded in the hierarchical RGB feature maps, and updated step-by-step in the encoding stage. Specifically, given the feature map of an RGB image in the *l*-th level $f_{l}^{I}$, and the updated depth feature map in the previous level $f_{l-1}^{D}$, the ADIM integrates the RGB-D multi-modalities as followins,

(2) $$f_{l}^{D},f_{l}^{A}=ADIM_{l}\left(f_{l-1}^{D},f_{l}^{I}\right),l=1,2,\cdots ,5$$

in which $f_{l}^{A}$ is the integrated feature map, $f_{l}^{D}$ is the updated depth feature, and $f_{0}^{D}$ is the depth image.

**Figure 3:**
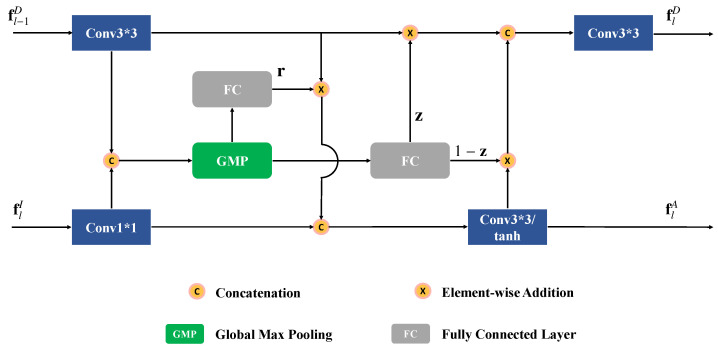
Structure of the ADIM.

The detailed structure of ADIM is shown in Figure 3. The implementation of ADIM is inspired by the gated recurrent unit (GRU), which is designed for dealing with sequential issues. We formulate the multi-scale feature integration process as a sequence problem and treat each scale as a time step. By this means, the ADIM iteratively updates the depth features of the previous state, and selectively fuses meaningful cues of two modalities with the memory mechanism.

In each time step, we feed image features $f_{l}^{I}$, into the GRU, and depth features $f_{l-1}^{D}$, can be regarded as the hidden state of the previous step. The output feature maps $f_{l}^{D}$ and $f_{l}^{A}$, in Equation (2), are the updated states. First, feature maps $f_{l}^{I}$ and $f_{l-1}^{D}$, are encoded by a convolutional layer and an ReLU activation layer, and the output feature maps are denoted as ${\tilde{f}}_{l}^{I}$ and ${\tilde{f}}_{l-1}^{D}$, respectively. Then the two feature maps are concatenated and transformed by a global max-pooling (GMP) operation, and a feature vector is generated. Subsequently, two separate fully connected layers, followed by the sigmoid function, are applied on the feature vector, to generate the resent gate r and the update gate z. In fact, gate r controls the integration of the depth and RGB features, and z controls the update step of $f_{l}^{D}$. Based on them, the fused multi-modal feature $f_{l}^{A}$, and the updated depth representation $f_{l}^{D}$, are output. Formally, the above process can be formulated as:

(3) $$\left\{\begin{array}{cc} r & =\sigma (FC_{\theta_{r}}\left(GMP\left(C\left({\tilde{f}}_{l-1}^{D},{\tilde{f}}_{l}^{I}\right)\right)\right))\\ z & =\sigma (FC_{\theta_{z}}\left(GMP\left(C\left({\tilde{f}}_{l-1}^{D},{\tilde{f}}_{l}^{I}\right)\right)\right))\\ f_{l}^{A} & =\tanh(C\left(r⊗{\tilde{f}}_{l-1}^{D},{\tilde{f}}_{l}^{I}\right))\\ f_{l}^{D} & =\sigma (Conv\left(C\left(z⊗{\tilde{f}}_{l-1}^{D},\left(1-z\right)⊗f_{l}^{A}\right)\right)) \end{array}\right.$$

in which $FC_{\theta }\left(\cdot \right)$ is the fully connected layer with learnable parameters θ, GMP(·) is the global max-pooling operation, and ⊗ is the channel-wise multiplication operation. The feature map $f_{l}^{A}$, generated by the hidden layer, memorizes valuable multi-modal information of previous scales, which is adaptively integrated with the features of the current scale. Such an operation enhances the interaction between cross-modalities as the network goes deeper. The depth feature $l^{D}$, is also updated according to the corresponding appearance information in $f_{l}^{A}$, and further facilitates the cross-modal learning in the next scale.

### 3.3. Relative Depth-Induced Module

Besides the absolute depth values, we can learn contrastive and structural information about the scene from relative depth values in the local area. Relative depth information can be extracted from the depth image and reveals the spatial relationship between pixels. Intuitively, closer pixels in the depth space should have a more compact feature interaction, as they tend to have the same saliency label. This observation is essential for separating salient objects in extremely cluttered image scenes. In this work, we proposd an RDIM that utilizes the GCN, to ensure message propagation, by using relative depth information. As shown in Figure 2, RDIMs are employed in the 3rd and 4th levels of the decoding stage. Given the feature map $f_{l}^{P}$, in Equation (1), and the depth image, the RDIM refines the integrated multi-scale feature maps to boost the performance of the saliency model.

(4) $$f_{l}^{R}=RDIM_{l}\left(f_{l}^{P},D\right)$$

**Graph Construction.** To explore the relative depth relationship between pixels, we represent the feature map $f_{l}^{P}$, generated as an undirected graph G=(V,E), with the node set *V*, and edge set *E*. First, the depth image is resized to the size of $f_{l}^{P}$. Then, each pixel in $f_{l}^{P}$ is regarded as a node in the graph, and the node set is denoted as $V=\{n_{1},n_{2},\cdots ,n_{K}\}$, where *K* is the total pixel number. Each node $n_{i}$, corresponds to a 3D coordinate $\left(x_{i},y_{i},d_{i}\right)$ and a feature vector $f_{l,i}^{P}$, where $\left(x_{i},y_{i}\right)$ is the 2D spatial coordinate in the feature map, $d_{i}$ is the depth value of the pixel, and $f_{l,i}^{P}$ is the feature vector on the channel dimension, of the *i*-th pixel in the feature map $f_{l}^{P}$.

To allow message transmission in the local area, we define edges between each node and their *m* nearest neighbors, according to their 3D coordinates. The weight on edge $e_{ij}\in E$, is defined as the relative depth value $w_{ij}=\left|\left(x_{i},y_{i},d_{i}\right)-\left(x_{j},y_{j},d_{j}\right)\right|$, to measure the spatial correlation between the nodes $n_{i}$ and $n_{j}$.

**Graph Convolutional Layer.** The proposed spatial GCN consists of a series of stacked graph convolutional layers (GCLs). For each GCL, we first define the semantic affinity $a_{ij}$, for the edge $e_{ij}$, to characterize the semantic discrepancy between nodes $n_{i}$ and $n_{j}$. Specifically, to further consider the global contextual information of the image, a global average pooling (GAP) operation is applied on the feature map $f_{l}^{P}$, to extract high-level semantic information, and the output feature vector is denoted as $f_{g}$. The semantic affinity is formulated as

(5) $$a_{ij}=FC_{\theta_{1}}\left(C\left(f_{l,i}^{P},f_{l,j}^{P},f_{g}\right)\right),$$

where $f_{l,i}^{P}$ and $f_{l,j}^{P}$ are feature vectors of $n_{i}$ and $n_{j}$, and $FC_{\theta_{1}}\left(\cdot \right)$ is the fully connected layer with learnable parameters $\theta_{1}$. Then the feature $f_{l,i}^{P}$ of each node $n_{i}$, is updated by the fully connected layer

(6) $${\tilde{f}}_{l,i}^{P}=FC_{\theta_{2}}\left(C\left(\sum_{j\in N(n_{i})}w_{ij}a_{ij},f_{l,i}^{P},f_{g}\right)\right),$$

where $N(n_{i})$ is the set of neighbors of the node $n_{i}$. In the updating process, both semantic and spatial affinities are considered, which helps to improve the discrimination ability of the feature.

In the RDIM, three GCLs are sequentially applied, to update the global semantic feature $f_{g}$, the semantic affinity $a_{ij}$, and the node feature ${\left\{{\tilde{f}}_{l,i}^{P}\right\}}_{i=1}^{N}$. We adopt the output feature of the last GCL as the refined one, and denote it as $f_{l,i}^{R}$. Note that, $f_{l,i}^{R}$ is the channel-wise feature vector in the location $\left(x_{i},y_{i}\right)$. We then re-arrange features of all nodes to form a feature map $f_{l}^{R}$, which has the same size as $f_{l}^{P}$. According to Equation (4), the feature map $f_{l}^{R}$, is the final output of the RDIM at scale *l*.

Intuitively, the constructed graph on feature map $f_{l}^{P}$, reveals the affinity between nodes in terms of both spatial correlation and visual association. By transferring messages between nodes using the GCN, the feature of each node is refined, according to its affinity with short-range neighbors. This encourages similar nodes (in both spatial and visual space) to have the same saliency labels.

The generated feature maps $f_{l}^{R}$, of the RDIM, are then input into the next decoding stage. To balance the computation costs and performance, we employ the RDIM in the 3rd and 4th scales in the decoding stage. We set *m* to be 64 in the 3rd scale and 32 in the 4th scale.

### 3.4. Training and Inference

To constrain the network and learn effective saliency cues, we generate a saliency map in each scale of the network and supervise it by the ground truth image. Specifically, the feature map $f_{l}^{P}\left(i=1,2,\cdots ,5\right)$ of the *l*-th level in the decoding stage, is transformed by a 3×3 convolutional layer, into one channel. This is followed by an up-sampling operation, to resize the output into the size of the input image, and we denote this result as the saliency map of the *l*-th level, $s_{l}$. This saliency prediction is supervised by the ground truth image $\hat{s}$, by the cross-entropy loss function

(7) $$L\left(s_{l},\hat{s}\right)=-\sum_{i,j}\hat{s}\left(i,j\right)\log\left(s_{l}\left(i,j\right)\right)+\left(1-\hat{s}\left(i,j\right)\right)\log\left(1-s_{l}\left(i,j\right)\right).$$

The total loss is the summation of the loss in each scale, and the proposed DIN is trained in an end-to-end manner, by minimizing the total loss.

Considering the saliency map $s_{1}$, generated by the feature map $f_{1}^{P}$, incorporates both multi-level and multi-modal information, we employ it as the final prediction result of the network.

## 4. Results and Analysis

### 4.1. Experiment Setup

**Implementation Details.** The parameters of the backbone network are fine-tuned by the pre-trained ResNet-50 [51], on the Imagenet [52] dataset. The rest of the parameters are randomly initialized. The input images are resized to 256×256, by the bilinear interpolation operation. We utilize the same data augmentation methods as in [35], to prevent the network from overfitting, including randomly flipping, cropping, and rotating. The Adam algorithm [53] is employed to optimize the loss function. The base learning rate is set to be $5\times 10^{-5}$ and is decreased by ten times every 20 epochs. Our network converges within 40 epochs. The weight decay is 0.0001, and the batch size is four. All experiments are implemented on the PyTorch platform, with a single NVIDIA GTX 2080Ti GPU. The model size of the proposed DIN is 99.55 Mb, and the speed is 11 FPS.

**Datasets.** We evaluate saliency models on six large-scale datasets, including **NLPR** [33], **NJUD** [34], STERE [54], **SIP** [55], **LFSD** [56], and **SSD** [57]. **NLPR** includes 1000 images, most of which contain multiple salient objects, and the corresponding depth images are captured by Kinect. **NJUD** incorporates 1985 images, which are collected from the internet, 3D movies, and photographs taken by a Fuji W3 stereo camera. **STERE** consists of 1000 pairs of binocular images. **SIP** includes 1000 images, with salient persons in real-world scenes. **LFSD** is composed of 100 light field images, which are taken by the Lytro light field camera. **SSD** contains 80 images that are extracted from stereo movies. As suggested in [35], we employ 1485 images from the **NJUD** and 700 images from the **NLPR** as the training set. The rest of the images are used as the test set.

**Evaluation Metrics.** We employ four metrics to evaluate the performance of the different algorithms, including max F-measure [58], structural measure (S-measure) [59], enhanced-alignment measure (E-measure) [60], and mean absolute error (MAE).

Saliency maps are first binarized by a set of thresholds ranging from 0 to 255, and compared with ground truth images. Then, precision and recall values are computed. **F-measure** considers both precision and recall values, to evaluate the performance of saliency models comprehensively. F-measure is defined as the weighted harmonic mean of the precision and recall,

(8) $$F_{\beta }=\frac{(1+\beta^{2})\times Precision\times Recall}{\beta^{2}\times Precision+Recall},$$

where β is set to 0.3 following [58]. **S-measure** evaluates the structure similarity between objects detected by saliency maps and those in ground truth images. It considers both object-aware similarity $S_{object}$, and region-aware similarity $S_{region}$, by a linear system,

(9) $$S_{\mu }=\mu \times S_{object}+\left(1-\mu \right)\times S_{region},$$

in which μ is the constant parameter that balances the importance of the object-aware and region-aware similarity. **E-measure** computes the enhanced alignment matrix $\phi_{FM}$, to capture the pixel-level matching and image-level statistics of the foreground map, and uses the mean value of the matrix $\phi_{FM}$, to reflect the quality of the saliency prediction,

(10) $$E_{FM}=\frac{1}{H\times W}\sum_{i,j}\phi_{FM}\left(i,j\right).$$

MAE computes the mean absolute difference between the prediction result s, and the ground truth image $\hat{s}$,

(11) $$MAE=\frac{1}{H\times W}\sum_{i,j}\left|s\left(i,j\right)-\hat{s}\left(i,j\right)\right|.$$

### 4.2. Evaluation with State-of-the-Art Models

We compare the proposed DIN model with 17 state-of-the-art saliency models, including DANet [36], PGAR [61], CMWNet [62], ATSA [19], D3Net [55], DSA2F [20], DCF [63], HAINet [64], CDNet [65], CDINet [66], MSIRN [67], SSP [39], FCMNet [68], and PASNet [44]. For a fair comparison, saliency maps of the evaluated methods are provided by the authors, or obtained by implementing the codes.

**Quantitative Evaluation.** We compare the proposed DIN model with the evaluated methods on six large-scale datasets. The max F-measure, S-measure, E-measure, and MAE values are demonstrated in Table 1. The quantitative experiments show that our method is able to achieve competitive performance against recent state-of-the-art algorithms on most datasets, demonstrating the effectiveness of the proposed method. Especially on the challenging **NJUD** [34] and **LFSD** [56] datasets, which incorporate semantically complicated images, the performance of our method is superior to other algorithms, indicating that the proposed DIN is able to learn informative semantic cues from cluttered scenarios.

**Figure 4:**
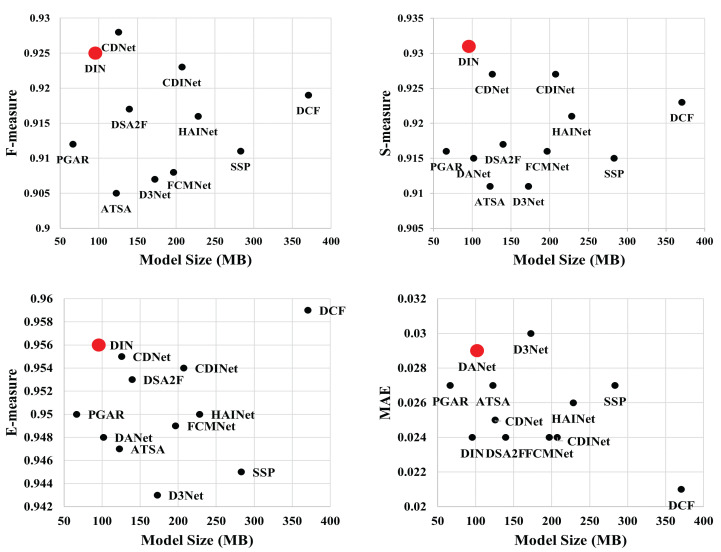
Scatter diagrams of model size and performance (F-measure, S-measure, E-measure, and MAE) on the **NLPR** dataset. The DIN achieves state-of-the-art performance with fewer parameters.

Table 2 shows the model size of the evaluated methods. The model size of the proposed DIN is much smaller than the comparison saliency models, except for the PGAR, which employs the VGG-16 [69] as the backbone network. Figure 4 illustrates the scatter diagrams of model size and performance of the evaluated methods in terms of F-measure, S-measure, E-measure, and MAE. The comparisons in Figure 4 intuitively demonstrate that DIN is able to achieve satisfactory performance with fewer parameters. This observation demonstrates a potential for the DIN to be deployed on mobile devices.

**Figure 5:**
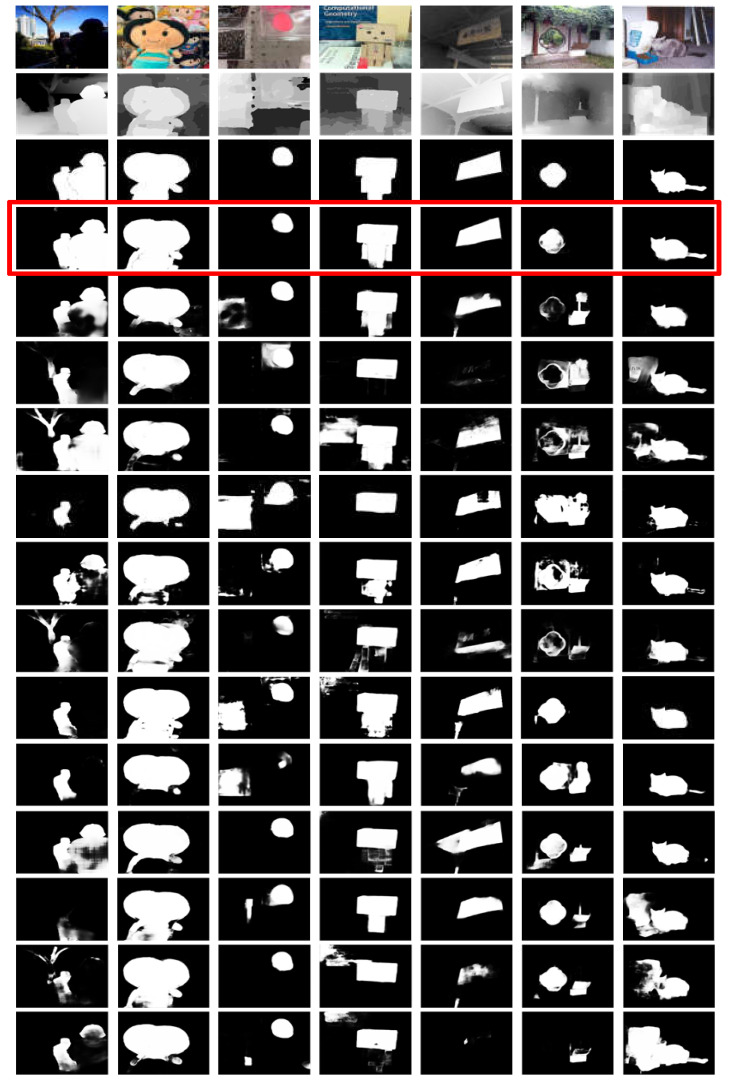
Visual examples of our method and the evaluated algorithms. From top to bottom: RGB image, depth image, GT, DIN (ours), DANet [36], PGAR [61], CMWNet [62], ATSA [19], D3Net [55], DSA2F [20], DCF [63], HAINet [64], CDNet [65], CDINet [66], and SSP [39].

**Qualitative Evaluation.** To qualitatively evaluate our method, we show some visual examples of our DIN model and other algorithms in Figure 5. Compared with other algorithms, our method is able to detect entire salient objects, with well-defined boundaries, accurately. As shown in Figure 5, the proposed DIN model is effective in various challenging scenarios, including multiple objects (1st column), salient objects with different colors (2nd column), distractors in the background (3rd, and 6–7th columns), low contrast between the foreground and background (5th column), and cluttered background (3rd, 4th, and 6–7th columns). For example, in the 1st column in Figure 5, most other algorithms cannot capture all salient regions of the two objects. In contrast, our method consistently highlights multiple salient objects. In the 2nd example, although there are various appearances in the foreground, the proposed DIN is able to detect all parts of salient regions. In the 3rd and 4th examples, our model successfully suppresses the distractors in the background regions. In the low contrast and low illumination scenarios (5th and 7th example), the proposed DIN can segment the entire salient regions from the background, because of the combination of the RGB and depth information. The foreground in the 6th example is not salient in the depth space. However, our method can still capture salient regions according to the contrast cues in the RGB and semantic space. The above visual results verify the effectiveness and superiority of the proposed DIN method against the comparison algorithms.

### 4.3. Ablation Studies

In this section, we demonstrate ablation studies, to verify the effectiveness of each main component of the proposed DIN model.

#### 4.3.1. Effectiveness of ADIM

The goal of the ADIM is to integrate absolution depth information and the RGB image, and explore complementary cues from the multi-modalities. In order to verify the effectiveness of the ADIM, two networks are trained for comparison:

**Baseline:** we utilize the backbone network as the baseline network, which replaces the ADIM as the concatenation and convolution block, and deletes the RDIM from the DIN. The baseline network takes the RGB and depth images as inputs and generates a final saliency map.

+ADIM: referred to as the +ADIM network, which takes both RGB and depth images as inputs, and employs ADIM to fuse multi-modal features hierarchically in the encoding stage based on the baseline network.

**Figure 6:**
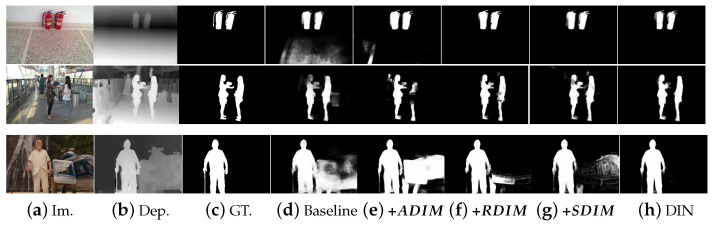
Visual effects of ablation studies. (**a**) Input image, (**b**) depth image, (**c**) ground truth, (**d**) baseline, (**e**) **+ADIM**, (**f**) **+RDIM**, (**g**) **+SDIM**, and (**h**) DIN.

As shown in Table 3 (1st and 2nd rows), **+ADIM** outperforms the baseline network by up to 3% on F-measure, 3% on S-measure, 4% on E-measure, and 32% on MAE, verifying the effectiveness of the proposed ADIM. Figure 6 shows visual results of the baseline and **+ADIM** network. In the 1st example in Figure 6, the baseline network captures two persons as salient objects (Figure 6d). However, the left one is not salient in both semantic and depth spaces. Thanks to the ADIM, the saliency map of the **+ADIM** network (Figure 6e) focuses more on the true object. In the 2nd example, the saliency map generated by the baseline network (Figure 6d) wrongly responds to the background regions. In contrast, the ADIM is able to alleviate this drawback by adaptively integrating the RGB and depth features, resulting in more accurate saliency prediction results (Figure 6e).

We also compare the proposed ADIM with existing cross-modal fusion modules, including the depth attention module (DAM) [19], cross reference Module (CRM) [63], and cross-enhanced integration module (CIM) [21], in the encoding stage. Specifically, we replace the ADIM in the **+ADIM** network, with DAM, CRM, and CIM, and denote the networks as **+DAM**, **+CRM**, and **+CIM**, respectively. Table 4 (b–d) show their performance in terms of F-measure, S-measure, E-measure, and MAE, respectively. By contrast, the performance of **+ADIM** in Table 4, is higher than the comparison modules by up to 1%, 1%, 2%, and 20%, on the four metrics, respectively. We attribute the increase to the message transmission of latent features across different scales, which is implemented by the GRU-based structure of the ADIM. In contrast, DAM, CRM, and CIM only focus on the information transfer between the two modalities.

#### 4.3.2. Effectiveness of RDIM

The RDIM aims to refine the multi-modal features by exploring the local contrast information indicated by relative depth values. To verify the effectiveness of the RDIM, we train **+RDIM** and **+SDIM** as baseline networks for comparison.

**+RDIM**: remove all ADIMs in the encoding stage of the DIN.

**+SDIM**: compared with the **+RDIM**, in the GCN, the weight on edge $e_{\mathrm{ij}}$ is defined as the spatial distance in 2D space, $w_{\mathrm{ij}}=\left|\left(x_{i},y_{i}\right)-\left(x_{j},y_{j}\right)\right|$.

As shown in Table 3, compared with the baseline network (1st row), the **+RDIM** (3rd row) achieves up to 3%, 3%, 4%, and 34% improvement on F-measure, S-measure, E-measure, and MAE, respectively. It is also superior to the **+ADIM** network (2nd row). This observation indicates that local contrast information is important for exploring detailed image cues and contributes more to obtaining accurate predictions. Figure 6 demonstrates the visual results of the **+RDIM** network. Compared with the saliency results of the baseline and **+ADIM** networks, those of the **+RDIM** (Figure 6f) present more accurate details around the edges of the objects. This is because the RDIM employs relative depth information to learn contrastive information in the local area, and improve the discriminative ability of multi-modal features by GCNs.

We also investigate the importance of relative depth values. In the **+SDIM** in Table 3, we construct the graph in the RDIM without considering the relative depth values. In other words, we only use the spatial distances of pair-wise pixels to reflect their affinities. According to the quantitative experiments in Table 3, the performance of **+SDIM** is lower than that of **+RDIM**, demonstrating the contribution of the relative depth values. The saliency detection results of the **+SDIM** in Figure 6g, also present cluttered noise around object boundaries and the background.

We compare the proposed RDIM with two existing modules in the decoding stage, including the multi-modal feature aggregation (MFA) module [21] and consistency-difference aggregation (CDA) module [39]. These two modules are applied to the decoding stage of the network, to further integrate and enhance the feature representation of multi-modalities. For a fair comparison, we replace the RDIM in the **+RDIM** network with the MFA and CDA modules, and denote them as **+MFA** and **+CDA**, respectively. Table 4 (f–g) demonstrate the quantitative performance of these networks. It can be seen that **+RDIM** outperforms the comparison networks by up to 1%, 1%, 2%, and 16% in terms of F-measure, S-measure, E-measure, and MAE, respectively. This is because the RDIM utilizes relative depth values to update the feature representation in consideration of its spatial and semantic affinities with other regions, modeling structural information of the scene, which is crucial for saliency prediction. By comparison, MFA and CDA only make use of absolute depth values and integrate them with RGB features, inevitably introducing clutter noise, because of the large gap between multi-modalities.

#### 4.3.3. Effectiveness of Combination of ADIM and RDIM

The DIN in Table 3 (5th row) is the proposed method in our work, which employs both ADIM and RDIM in the network. As shown in quantitative results, the DIN achieves the best performance compared with all of the other evaluated baseline networks, which indicates that the ADIM and RDIM are complementary.

Compared with the saliency maps of the baseline, **+ADIM**, and **+RDIM** networks (Figure 6d–f), the prediction results generated by the DIN in Figure 6g suppress the responses on cluttered background regions and accurately capture entire salient objects, with well-defined boundaries. In the 4th example in Figure 6, the baseline network (Figure 6d) is confused by the image background regions, and outputs blurry prediction results. The **+ADIM** (Figure 6e) captures both the man and helicopter as salient objects, since they are salient in the depth space. The **+RDIM** (Figure 6f) can alleviate the negative predictions on the image background by exploring the local contrast in both RGB and depth spaces. However, the prediction result is still inaccurate compared with the ground truth. Due to the reasonable interaction between ADIM and RDIM, the DIN (Figure 6g) can accurately capture the true salient object and eliminate the incorrect responses in the background regions. This observation verifies that the DIN takes full advantage of the complementary nature between the absolute and relative depth information and thus achieves satisfactory performance.

### 4.4. Failure Cases

**Figure 7:**
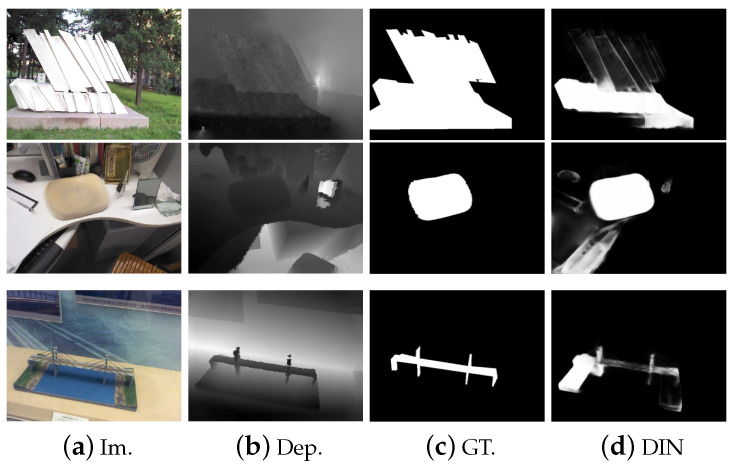
Failure cases of our method. (**a**) Input image; (**b**) depth image; (**c**) ground truth; (**d**) the proposed DIN.

Figure 7 shows some failure cases of our method. In the first example, the salient object has low-depth contrast with the surroundings. In the second example, the depth values of the salient object are not correctly captured by the sensor. The salient object in the third example is hard to recognize in the RGB space. Our proposed DIN fails to detect the true objects with clear boundaries in these scenarios. This is because, in these situations, depth maps do not provide valuable information and even introduce noisy responses, which limit the accuracy of the saliency model. Moreover, the RDIM based on relative depth values will magnify prediction errors.

## 5. Conclusions

In this work, we propose a DIN for RGB-D salient object detection. The DIN consists of two main components, ADIM and RDIM. The ADIM utilizes a GRU-based method, to successively integrate the RGB and absolute depth values at multiple scales. In the RDIM, we propose a spatial GCN, to explore detailed saliency cues with the help of relative depth values and semantic relationships. These two modules are complementary and lead to an effective saliency model which is able to detect entire salient objects with well-defined boundaries. The DIN is a lightweight model, because of the single-stream architecture. Extensive experiments show that the performance of the DIN is competitive with the state-of-the-art algorithms on six large-scale benchmarks. In future work, we will improve the robustness of the ADIM and RDIM in complex scenarios, and extend the proposed models to other cross-modal tasks. Moreover, we will explore more possibilities of the RGB-D salient object detection on engineering applications. 

## Figures and Tables

**Table 1:** Quantitative results of the evaluated methods in terms of the F-measure ($F_{max}$), S-measure ($S_{\mu }$), E-measure ($E_{\beta }$), and MAE (ϵ) on six datasets. The top three scores of each metric are marked as red, green, and blue. Higher scores of F-measure, S-measure, and E-measure are better, and lower scores of MAE are better.

Model	Pub.	NLPR [33]				NJUD [34]				STERE [54]			
		$F_{\max}$	$S_{\mu }$	$E_{\beta }$	ϵ	$F_{\max}$	$S_{\mu }$	$E_{\beta }$	ϵ	$F_{\max}$	$S_{\mu }$	$E_{\beta }$	ϵ
DANet [36]	20ECCV	0. 913	0.915	0.948	0.029	0.898	0.892	0.912	0.049	0.897	0.892	0.915	0.048
PGAR [61]	20ECCV	0.912	0.916	0.950	0.027	0.918	0.909	0.934	0.042	0.902	0.894	0.932	0.045
CMWNet [62]	20ECCV	0.913	0.917	0.940	0.029	0.913	0.903	0.923	0.046	0.911	0.905	0.930	0.043
ATSA [19]	20ECCV	0.905	0.911	0.947	0.027	0.904	0.887	0.927	0.047	0.910	0.898	0.942	0.038
D3Net [55]	21TNNLS	0.907	0.911	0.943	0.030	0.910	0.900	0.914	0.047	0.904	0.899	0.921	0.046
DSA2F [20]	21CVPR	0.917	0.917	0.953	0.024	0.919	0.903	0.937	0.039	0.910	0.898	0.942	0.039
DCF [63]	21CVPR	0.919	0.923	0.959	0.021	0.923	0.911	0.944	0.035	0.911	0.901	0.942	0.039
HAINet [64]	21TIP	0.916	0.921	0.950	0.026	0.919	0.909	0.917	0.039	0.918	0.909	0.929	0.039
CDNet [65]	21TIP	0.928	0.927	0.955	0.025	0.925	0.915	0.943	0.037	0.917	0.909	0.942	0.038
CDINet [66]	21ACMMM	0.923	0.927	0.954	0.024	0.928	0.918	0.944	0.036	0.904	0.899	0.939	0.043
MSIRN [67]	21EAAI	0.918	0.931	0.962	0.025	0.913	0.912	0.942	0.038	0.780	0.904	0.936	0.045
SSP [39]	22AAAI	0.911	0.915	0.945	0.027	0.912	0.903	0.908	0.043	0.897	0.886	0.919	0.048
FCMNet [68]	22NeuCom	0.908	0.916	0.949	0.024	0.907	0.901	0.929	0.044	0.904	0.899	0.939	0.043
PASNet [44]	22CVPRW	0.921	0.913	0.966	0.021	0.892	0.867	0.938	0.051	-	-	-	-
DIN	Ours	0.925	0.931	0.956	0.024	0.925	0.920	0.945	0.035	0.915	0.909	0.932	0.038
**Model**	**Pub.**	**SIP** [55]				**LFSD** [56]				**SSD** [57]			
		$F_{\max}$	$S_{\mu }$	$E_{\beta }$	ϵ	$F_{\max}$	$S_{\mu }$	$E_{\beta }$	ϵ	$F_{\max}$	$S_{\mu }$	$E_{\beta }$	ϵ
DANet [36]	20ECCV	0.900	0.878	0.916	0.055	0.871	0.845	0.878	0.082	0.887	0.869	0.907	0.051
PGAR [61]	20ECCV	0.852	0.838	0.889	0.073	0.835	0.816	0.870	0.091	0.821	0.832	0.883	0.068
CMWNet [62]	20ECCV	0.889	0.867	0.908	0.062	0.899	0.876	0.908	0.067	0.883	0.875	0.902	0.051
ATSA [19]	20ECCV	0.884	0.851	0.899	0.064	0.883	0.854	0.897	0.071	0.867	0.852	0.916	0.052
D3Net [55]	21TNNLS	0.881	0.860	0.902	0.063	0.840	0.825	0.853	0.095	0.861	0.857	0.897	0.059
DSA2F [20]	21CVPR	0.891	0.861	0.911	0.057	0.903	0.882	0.923	0.054	0.878	0.876	0.913	0.047
DCF [63]	21CVPR	0.899	0.875	0.921	0.052	0.867	0.841	0.883	0.075	0.868	0.864	0.905	0.049
HAINet [64]	21TIP	0.915	0.886	0.924	0.049	0.880	0.859	0.895	0.072	0.864	0.861	0.904	0.053
CDNet [65]	21TIP	0.907	0.879	0.919	0.052	0.898	0.878	0.912	0.063	0.871	0.875	0.922	0.046
CDINet [66]	21ACMMM	0.903	0.875	0.912	0.055	0.890	0.870	0.915	0.063	0.867	0.853	0.906	0.056
MSIRN [67]	21EAAI	0.884	0.879	0.920	0.056	0.863	0.862	0.898	0.077	0.868	0.880	0.918	0.050
SSP [39]	22AAAI	0.895	0.869	0.909	0.059	0.870	0.853	0.886	0.076	0.887	0.882	0.921	0.042
FCMNet [68]	22NeuCom	0.883	0.862	0.903	0.068	0.860	0.855	0.903	0.055	0.881	0.858	0.912	0.062
PASNet [44]	22CVPRW	0.956	0.936	0.987	0.016	-	-	-	-	-	-	-	-
DIN	Ours	0.910	0.880	0.925	0.048	0.900	0.880	0.913	0.066	0.887	0.877	0.920	0.044

**Table 2:** Model size of the evaluated methods.

Model	DANet	PGAR	ATSA	D3Net	DSA2F	DCF
Model Size (Mb)	101.77	66.53	122.79	172.53	139.48	370.47
Model	HAINet	CDNet	CDINet	SSP	FCMNet	DIN
Model Size (Mb)	228.32	125.65	207.40	283.11	196.65	95.55

**Table 3:** Ablation studies in terms of F-measure, S-measure, E-measure, and MAE on **NLPR**, **NJUD**, and **STERE** datasets.

Model	NLPR				NJUD				STERE			
	$F_{\max}$	$S_{\mu }$	$E_{\beta }$	ϵ	$F_{\max}$	$S_{\mu }$	$E_{\beta }$	ϵ	$F_{\max}$	$S_{\mu }$	$E_{\beta }$	ϵ
**Baseline**	0.888	0.893	0.909	0.041	0.887	0.879	0.898	0.061	0.880	0.877	0.900	0.063
**+ADIM**	0.906	0.917	0.943	0.028	0.910	0.905	0.929	0.043	0.902	0.890	0.922	0.050
**+RDIM**	0.915	0.920	0.949	0.027	0.917	0.909	0.935	0.041	0.910	0.900	0.927	0.048
**+SDIM**	0.910	0.913	0.944	0.031	0.912	0.902	0.927	0.044	0.904	0.893	0.922	0.051
**DIN**	**0.925**	**0.931**	**0.956**	**0.024**	**0.925**	**0.920**	**0.945**	**0.035**	**0.915**	**0.909**	**0.932**	**0.038**

**Table 4:** Comparisons of ADIM and RDIM with other multi-modal fusion modules in the encoding and decoding stages, respectively.

Model	$F_{\max}$	$S_{\mu }$	$E_{\beta }$	ϵ
(a) **Baseline**	0.888	0.893	0.909	0.041
(b) **+DAM**	0.897	0.910	0.923	0.035
(c) **+CRM**	0.903	0.912	0.931	0.033
(d) **+CIM**	0.903	0.910	0.934	0.030
(e) **+ADIM**	**0.906**	**0.917**	**0.943**	**0.028**
(f) **+MFA**	0.909	0.912	0.933	0.032
(g) **+CDA**	0.910	0.914	0.937	0.031
(h) **+RDIM**	**0.915**	**0.920**	**0.949**	**0.027**
(i) **DIN**	**0.925**	**0.931**	**0.956**	**0.024**

## Data Availability

Data sharing not applicable.
